# Detection of high levels of mutations involved in anti-malarial drug resistance in *Plasmodium falciparum *and *Plasmodium vivax *at a rural hospital in southern Ethiopia

**DOI:** 10.1186/1475-2875-10-214

**Published:** 2011-08-02

**Authors:** Patricia Mula, Amalia Fernández-Martínez, Aida de Lucio, Jose Manuel Ramos, Francisco Reyes, Vicenta González, Agustín Benito, Pedro Berzosa

**Affiliations:** 1 National Centre for Tropical Medicine, Institute of Health Carlos III. C/Sinesio Delgado 4-6, 28029, Madrid, Spain; 2 Gambo General Rural Hospital, Shashemane, Ethiopia; 3 Infectious Diseases Unit, Hospital General Universitario de Elche, Alicante, Spain

## Abstract

**Background:**

In Ethiopia, malaria is caused by *Plasmodium falciparum *and *Plasmodium vivax*, and anti-malarial drug resistance is the most pressing problem confronting control of the disease. Since co-infection by both species of parasite is common and sulphadoxine-pyrimethamine (SP) has been intensively used, resistance to these drugs has appeared in both *P. falciparum *and *P. vivax *populations. This study was conducted to assess the prevalence of anti-malarial drug resistance in *P. falciparum *and *P. vivax *isolates collected at a rural hospital in southern Ethiopia.

**Methods:**

A total of 1,147 patients with suspected malaria were studied in different months across the period 2007-2009. *Plasmodium falciparum dhfr *and *dhps *mutations and *P. vivax dhfr *polymorphisms associated with resistance to SP, as well as *P. falciparum pfcrt *and *pfmdr1 *mutations conferring chloroquine resistance, were assessed.

**Results:**

PCR-based diagnosis showed that 125 of the 1147 patients had malaria. Of these, 52.8% and 37.6% of cases were due to *P. falciparum *and *P. vivax *respectively. A total of 10 cases (8%) showed co-infection by both species and two cases (1.6%) were infected by *Plasmodium ovale*. *Pfdhfr *triple mutation and *pfdhfr/pfdhps *quintuple mutation occurred in 90.8% (95% confidence interval [CI]: 82.2%-95.5%) and 82.9% (95% CI: 72.9%-89.7%) of *P. falciparum *isolates, respectively. *Pfcrt *T76 was observed in all cases and *pfmdr1 *Y86 and *pfmdr1 *Y1246 in 32.9% (95% CI: 23.4%-44.15%) and 17.1% (95% CI: 10.3-27.1%), respectively. The *P. vivax dhfr *core mutations, N117 and R58, were present in 98.2% (95% CI: 89.4-99.9%) and 91.2% (95% CI: 80.0-96.7%), respectively.

**Conclusion:**

Current molecular data show an extraordinarily high frequency of drug-resistance mutations in both *P. falciparum *and *P. vivax *in southern Ethiopia. Urgent surveillance of the emergence and spread of resistance is thus called for. The level of resistance indicates the need for implementation of entire population access to the new first-line treatment with artemether-lumefantrine, accompanied by government monitoring to prevent the emergence of resistance to this treatment.

## Background

Malaria is the vector-borne disease with the highest impact on the world's human population. In 2008, there were an estimated 243 million cases, leading to nearly 863,000 malaria-related deaths [1]. Although malaria-endemic areas are mainly restricted to tropical and subtropical regions at present, several models nonetheless project the geographical expansion of potential malaria transmission over the next few decades, along with more substantial changes later in the century [2]. Approximately 90% of clinically manifest infections of and practically all deaths from malaria are caused by *Plasmodium falciparum*. Ranking second in importance is *Plasmodium vivax*, which accounts for nearly 10% of global malaria incidence. Roughly 90% of all malaria cases occur in tropical Africa [3].

Approximately 52 million people in Ethiopia are considered to be at risk of the disease [4]. While 4 to 6 million clinical malaria cases are annually reported by the country's health facilities, the real number is estimated to be as high as 10 to 15 million [5]. The major *Plasmodium *species causing malaria in Ethiopia are *P. falciparum *(about 60% of cases) and *P. vivax *(about 40% of cases), with the former being the cause of the most severe clinical manifestations and most deaths [6-8]. Malaria transmission follows a seasonal pattern (September-November), depending on the altitude and rainy season [7,9]. Epidemic malaria is frequent [10], particularly in the highlands (1,000-2,000 m above sea level).

Anti-malarial drug resistance in *P. falciparum *and *P. vivax *is the most pressing problem confronting malaria control in many endemic countries [11,12]. *Plasmodium falciparum *has developed resistance to a series of drugs, and *P. vivax *is resistant to chloroquine (CQ) and not primaquine-tolerant (PQ) [13,14]. Furthermore, in countries where intensive use has been made of sulphadoxine-pyrimethamine (SP), resistance to these drugs has appeared in *P. vivax *populations [15-20], though some authors view this as being a sign of innate resistance to sulphadoxine in such parasites [21].

Current studies report high rates of therapeutic failure (72%) in some areas of Ethiopia [22], due to the presence of SP resistance in the two principal species. In 2004, artemether-lumefantrine (AL) (Coartem^®^) was introduced into the country as a first-line treatment, and the national guidelines have since prescribed this drug for uncomplicated malaria [4]. Even so, not everyone is benefiting from this treatment because 85% of the population live in rural areas where access to basic health care is severely limited.

In general, the presence of high resistance to SP has stopped it from being used as a treatment in African endemic countries, and so nowadays it is only used, in combination with insecticide-treated mosquito nets, for Intermittent Preventive Treatment (IPT) in pregnant women and in areas where it has been given to children under five years of age has provided encouraging results [23].

The study of point mutations (Single Nucleotide Polymorphisms - SNPs) in molecular markers is an extremely useful epidemiological tool, which enables the emergence and spread of mutant parasites to be monitored. Analyses of different molecular markers of resistance are currently used, namely: the T76 point mutation in the *P. falciparum pfcrt *gene; the Y86, F184, C1034, N1042 and Y1246 point mutations in the *pfmdr1 *gene [24]; the L164, N/T108, I51 and R59 point mutations in the *pfdhfr *gene; and the G437 and G581, A/F436, E540 and S613 point mutations in the *pfdhps *gene [12,24-28].

*Plasmodium vivax *resistance to SP is associated with mutations that appear in the homologous genes, *pvdhfr *(N117, R58 and T117) and *pvdhps *(G383, G553) [17,18]. Mutations in *pvdhfr *have been implicated in resistance to pyrimethamine *in vivo *[29]. Furthermore, it seems that *pvdhfr *mutations at residues 117 and 58 have been observed to arise first when drug pressure is applied [17,18,30]. The double and triple mutations, N117/R58 and N117/R58/L57, are associated with delayed parasite clearance following SP treatment [17] and with therapeutic failure in many regions of south-east Asia [17,31,32], Iran [30], Pakistan [33], India [34], Colombia [28] and Madagascar [35].

However, the appearance of different combinations of L57/R58/N117/G383/G553 mutations in both the *pvdhfr *and *pvdhps *genes has been associated with SP treatment failure only in patients infected by *P. vivax *and not in those co-infected by both species [36,37] in which no mutations have been found in the orthologous genes of *P. falciparum*.

This study sought to determine the following: the prevalence of *P. falciparum *and *P. vivax *in patients with clinically suspected malaria, attending the Gambo General Rural Hospital in Ethiopia; and the prevalence of mutations in genes involved in resistance to different anti-malarial drugs, based on samples collected in different months across the period 2007-2009.

## Methods

### Study site and biological samples

**Figure 1:**
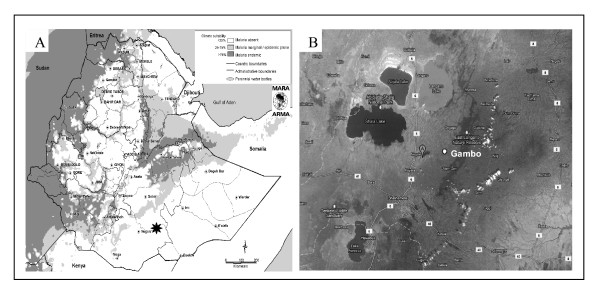
**Gambo location in Ethiopia**. The left figure (1 A) displays the location of Gambo on the map of Ethiopia (black star). The right figure (1 B) shows the satellite image of Gambo. Source of maps: 1 A: http://www.mara.org.za/; 1 B: Google Maps.

The Gambo General Rural Hospital is situated in the province of West Arsi, Ethiopia, 245 kilometres south-east of the capital, Addis Ababa (Figure 1), at an altitude of 2,250 metres (approximately 7,382 feet) above sea level. The hospital provides daily health care and covers nine peripheral health centres serving 23 villages, with a total population of 98,000. Patients may come from bordering areas, lying at lower altitudes.

The study was conducted during different calendar months across the period 2007-2009. At the hospital, a total of 1,147 blood samples were collected from patients (age range, 1 month to 75 years) with clinically suspected malaria (see Table 1). Two capillary blood specimens were taken from each patient, one for confirmation of *Plasmodium *infection by microscopic observation of thin and thick Giemsa-stained blood films, and the other on filter paper (Whatman^© ^3 MM) for molecular assay.

**Table 1:** Number of samples collected by year of study.

Year	Months	No of Months	Samples
2007	Nov-Dec	2	175
2008	Jan-May	5	432
2009	Feb-May	4	540

		Total months: 11	Total samples: 1,147

(Nov: November/Dec: December/Jan: January/Feb: February).

DNA extraction was performed using commercial kits (Speedtools tissue DNA Extraction Kit, Biotools), and *Plasmodium *species were subsequently confirmed by Seminested-Multiplex PCR [38]. The nested PCR-RFLP technique was used to analyse: codons 76 of *pfcrt*, 86 and 1246 of *pfmdr1*, 51, 59, 108 and 164 of *pfdhfr *and 436, 437, 540 and 581 of *pfdhps *from samples infected by *P. falciparum *[39]; and codons *pvdhfr *57, 58 and 117 and *pvdhps *383 and 553 from samples infected with *P. vivax*. The laboratory conditions and restriction enzymes have been described elsewhere [17,18,30]. As in other resistance studies [40], genes that showed mixed wild-type and mutant alleles were deemed to be mutant only when the amplification fragment was more intense for the latter. The study protocol was approved by the research review boards of the Institute of Health Carlos III (Madrid, Spain) and Gambo General Rural Hospital. Study participants with microscopy-confirmed malaria infection were treated in accordance with Ethiopian national guidelines [4,5]. A descriptive analysis was performed and estimates of prevalence along with their 95% confidence intervals (CIs) were obtained, using the method described by Newcombe [41].

## Results

### Molecular results

The presence of parasites was confirmed by Seminested-Multiplex-PCR in all the samples. Of the 1,147 samples, a total of 125 (10.9%; 95% CI: 9.2-12.8%) were shown to be infected by *Plasmodium sp*., which broke down as follows: *P. falciparum*, 66 (5.8%; 95% CI: 4.5-7.3%); *P. vivax*, 47 (4.1%; 95% CI: 3.1-5.5%); *P. falciparum *and *P. vivax *combined, 10 (0.9%; 95% CI: 0.4-1.6%); and *Plasmodium ovale*, 2 (0.2%; 95% CI: 0.03-0.7%). The prevalence of *Plasmodium *species in infected cases (n = 125) is shown in Table 2.

**Table 2:** Prevalence of *Plasmodium *species in infected patients (n = 125).

Infection	n = 125	Frequency (%)
*P. falciparum*	66	52.8%
*P. vivax*	47	37.6%
*P. ovale*	2	1.6%
Mixta *P. falciparum/P. vivax*	10	8.0%

**Figure 2:**
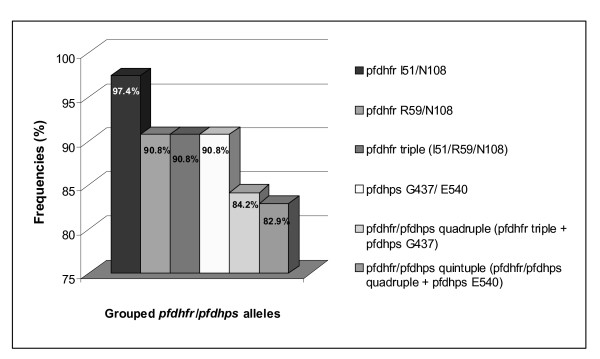
**Prevalence of grouped *pfdhfr/pdhps *mutant alleles in *P. falciparum *linked to sulphadoxine-pyrimethamine treatment failure (n = 76)**.

The frequencies of mutations and the combinations of these in the genes involved in anti-malarial drug resistance in *P. falciparum *are shown in Table 3 and Figure 2 respectively. Whereas all samples infected with *P. falciparum *presented with the *pfcrt *T76 mutation (95 CI: 95.1-100%), 32.9% (95% CI: 23.4-44.1%) and 17.1% (95% CI: 10.3-27.1%) of samples infected with *P. falciparum *had the *pfmdr1 *Y86 and *pfmdr1 *Y1246 mutations respectively.

**Table 3:** Prevalence of mutations conferring resistance to chloroquine and sulphadoxine-pyrimethamine in *Plasmodium falciparum *(n = 76).

Gene Locus	n = 76	Mutation (%)
*pfcrt *76	76	(100%)
*pfmdr1 *86	25	(32.9%)
*pfmdr1 *1246	13	(17.1%)
*pfdhfr *51	74	(97.4%)
*pfdhfr *59	69	(90.8%)
*pfdhfr *108	76	(100%)
*pfdhfr *164	1	(1.3%)
*pfdhps *436	14	(18.4%)
*pfdhps *437	70	(92.1%)
*pfdhps *540	52	(68.4%)
*pfdhps *581	11	(14.5%)

As with *pfcrt *T76, all samples infected with *P. falciparum *showed a 100% prevalence of mutation at *pfdhfr *N108. Only one case (1.3%; 95% CI: 0.2-7.1%), however, presented with the mutation at *pfdhfr *L164. The *pfdhfr *I51 and R59 mutations, responsible for modulating resistance to pyrimethamine, appeared in 97.4% (95% CI: 90.9-99.3%) and 90.8% (95% CI: 82.2-95.5%) of cases respectively. The triple I51/R59/N108 mutation was observed in over 90% (95% CI: 82.2-95.5%) of cases (Figure 2).

Insofar as the SNPs in *pfdhps *were concerned, none of the samples had wild genotypes at the codons studied. Both *pfdhps *A436 and *pfdhps *G581 mutations appeared at low frequencies (18.4% [95% CI: 11.3-28.9%] and 14.5%, [95% CI: 8.3-24.1%] respectively) compared to the *pfdhps *G437 and *pfdhps *E540 mutations, which appeared in 92.1% (95% CI: 83.8-96.3%) and 68.4% (95% CI: 57.3-77.8%) of cases respectively. The *pfdhps *double mutation, G437/E540, appeared in 84.2% (95% CI: 74.4-90.7%) of cases.

The *pfdhfr/pfdhps *quadruple and quintuple mutations, N108/I51/R59+G437 and N108/I51/R59+G437/E540 (both associated with therapeutic failure of SP), displayed a prevalence of over 80% (95% CI: 70-87.7%) among cases infected by *P. falciparum *(Figure 2).

While 98.2% (95% CI: 89.4-99.9%) of samples infected with *P. vivax *showed the N117 mutation at codon 117 of the *pvdhfr *gene, R58 appeared at codon 58 in 91.2% (95% CI: 80.0-96.7%) of cases. The *pvdhfr *double mutation, N117/R58, was observed in most cases of *P. vivax *infection (91.2%; 95% CI: 80.0-96.7%) but no case displayed mutation at codon 57 (95% CI: 0-7.9%).

With respect to the *pvdhps *gene, mutation was found at codon 553, with a prevalence of 3.5% (95% CI: 0.6-13.2%) and appeared at the same frequency (3.5%; (95% CI: 0.6-13.2%) in combination with the double N117/R58 mutation (Table 4).

**Table 4:** Prevalence of mutations conferring resistance to sulphadoxine-pyrimethamine in *Plasmodium vivax *(n = 57).

Gene Locus	N = 57	Mutation (%)
*pvdhfr *R58	52	(91.2%)
*pvdhfr *L57	0	(0%)
*pvdhfr *N117	56	(98.2%)
*pvdhfr *T117	0	(0%)
*pvdhfr *R58/N117	52	(91.2%)
*pvdhfr *R58/N117/L57	0	(0%)
*pvdhps *G383	0	(0%)
*pvdhps *G553	2	(3.5%)
*pvdhfr/pvdhps *R58/N117/G553	2	(3.5%)

All cases that were co-infected by both species (n = 10) and presented with mutations in *pfdhfr/pfdhps *(I51/R59/N108, 80% [95% CI: 49.0-94.3%]; N108/I51/R59+G437, 80% [95% CI: 49.0-94.3%]; and N108/I51/R59+G437/E540, 70% [95CI: 39.7-89.2]), also introduced the double mutation, N117/R58, (100%; 95% CI: 72.3-100%) in *pvdhfr*.

## Discussion

In areas where *P. falciparum *and *P. vivax *co-exist, parasite-specific diagnosis and choice of effective treatment is crucial to prevent the emergence and spread of resistance. In malaria-endemic areas, the occurrence of *P. falciparum/P. vivax *co-infection is frequent and it is, therefore, common for *P. vivax *to have been exposed to treatment with SP.

In Ethiopia, not only are there wide interregional differences in the endemicity and transmission of malaria, but there is also a significant lack of information on the effectiveness of anti-malarial drugs. In 1999, the CQ treatment failure rate in the first two weeks still stood at 88% in the centre of the country [42]. In 2005, SP therapeutic failure within two to four weeks of follow-up was 36% and 72% respectively. In this same year, however, treatment with AL yielded an appropriate clinical and parasitological response of 99% [22].

With regard to the prevalence of species, a decrease of *P. falciparum *mono-infection and an increase of *P. vivax *mono-infection was observed in contrast to other results obtained by other studies previously conducted in Ethiopia [6,7,43,44]. The predominant species was *P. falciparum*, which appeared in approximately 53% of cases, followed by *P. vivax *with a prevalence higher than 30%, and a small proportion cases of co-infection by both species. In addition, two cases of infection by *Plasmodium ovale *were detected. This finding is rather singular; in that *P. ovale *has never been previously described in Ethiopia, and might be accounted for by the migration process from the western side of the continent to this country, or people returning after having gone to work in other areas of Africa with prevalence of *P. ovale*. In the future, more studies about the prevalence of malaria species should be performed in Ethiopia, to know the factors are influencing in these changes of prevalence.

As with other studies in which high rates of therapeutic failure of CQ and SP have been detected, our results likewise show a high prevalence of these mutations linked to resistance in *P. falciparum*, particularly T76 in *pfcrt *(responsible for resistance to CQ) and the triple mutation in *pfdhfr *(responsible for treatment failure in SP) (see Table 3 and Figure 2). Furthermore, the high rate of the *pfdhps *double mutation, G437/E540, responsible for conferring a high degree of resistance to sulphadoxine and for therapeutic failure to SP in the presence of the triple mutation in *pfdhfr*, has resulted in the *pfdhfr/pfdhps *quintuple mutation being present in over 80% of cases. This corresponds to the low SP-treatment efficacy rates registered in different regions of Ethiopia [22] and confirms the need for AL to be made available countrywide as the first-line treatment.

The *pfcrt *T76, *pfmdr1 *Y86 and *pfmdr1 *Y1246 mutations are very useful molecular markers of CQ resistance in areas where resistance rates are low to mild [45,46]. This study showed high to moderate prevalence of *pfcrt *and *pfmdr1 *mutations, respectively, to be consistent with low CQ efficacy in Ethiopia [42].

In the case of *P. vivax*, there was a high prevalence of the *pvdhfr *R58 and *pvdhfr *N117 mutations. Furthermore, the *pvdhfr *double mutation, N117/R58, appeared in 52 out of 57 cases infected by *P. vivax *(taking the cases of *P. falciparum *co-infection into account), and 42 out of 47 cases infected by this species alone. The *pvdhps *G553 mutation always appeared in combination with *pvdhfr *double mutation R58/N117, resulting *pvdhfr/pvdhps *R58/N117/G553 mutation in two cases of *P. vivax *mono-infection. This finding could explain a possible SP treatment failure in these patients, as has been associated in other studies [36,37]. Yet *pvdhfr *T117, L57, *pvdhfr *triple or *pvdhfr/pvdhps *quadruple mutations were not in evidence, in the last case neither in *P. vivax *mono-infection nor in *P. vivax-P. falciparum *co-infection. Although these multiple mutations seem to be necessary for *in vivo *resistance [17,18,43], this finding nevertheless suggests widespread SP use, since the *pvdhfr *mutations arise first under drug pressure [17,18,43].

It should be taken into account that, when it comes to combined *P. falciparum/P. vivax *infection, patients should be treated with a blood schizonticide (for both species) and a tissue schizonticide (for hepatic hypnozoites of *P. vivax*). In the past, SP was used as treatment for *P. falciparum*. As a result, *P. vivax *came into contact with the same treatment when it was present in mixed infections, and mutations in different genes linked to SP resistance have thus appeared in this species. The mutations in the *pvdhps *gene would be equally accounted for, i.e., here, only the G553 mutation, implicated in resistance to sulphadoxine and corresponding to the G581 mutation in *P. falciparum*, displayed a very low prevalence and appeared in the presence of the *pvdhfr *double mutation, N117/R58. In contrast, the G383 mutation, which corresponds to G437 in *P. falciparum*, was observed in neither case. This strengthens the hypothesis that the asymmetric selection process of mutations observed in *P. falciparum *is also applicable to *P. vivax *[21].

Samples co-infected by these two species showed mutations in either *pfdhfr/pfdhps *or *pvdhfr*, which would explain why treatment for both these species of *Plasmodium *could fail in such patients.

In view of the genotype results obtained, it would be advisable for a study of therapeutic efficacy to be conducted in this area, to ascertain whether the genotypic are the same as the phenotypic data, i.e., whether the parity mutation-resistance is produced *in vivo*.

Moreover, it would be advisable for future research to verify whether there were mutations in genes (such as *atp6*) related with resistance to artemisinin derivatives, the use of which as an anti-malarial drug has spread in Africa. Artemisinin-based combination therapy (ACT) (for example, using Coartem^®^) has come into use in Ethiopia, though it is proving difficult to implement in rural areas due to the continued use of drugs, such as SP and CQ, in combination with other medication, a practice that continues to favour the expansion of resistance.

## Conclusions

1. The prevalence of *Plasmodium *species is slightly different from the results obtained by other authors in previous studies conducted in Ethiopia, showing an increase of *vivax *malaria cases and the presence of *P. ovale *for the first time.

2. The prevalence of mutations linked to CQ and SP resistance detected in *P. falciparum *and *P. vivax *in southern Ethiopia is higher than expected.

3. Implementation of permanent surveillance of resistance molecular markers as an epidemiological tool will enable health professionals and authorities to be alerted to the possible emergence and spread of resistance to new lines of treatment, such as ACT.

4. It is essential that the entire population be provided with access to new first-line treatment with ACT, which should be given under government supervision to avoid the drug being mismanaged and so prevent the emergence of any new resistances.

## Competing interests

The authors declare that they have no competing interests.

## Authors' contributions

PM was involved in the molecular studies, interpretation of results and drafted the manuscript. AFM and VG helped to do molecular studies. AL helped to do molecular studies and carried out sample collection. JMR performed the statistical analysis and interpretation, and helped to draft the manuscript. FR helped to collect the samples in Gambo (Ethiopia). AB helped to draft the manuscript and PB conceived and funded the project, carried out sample collection, and drafted the manuscript. All authors read and approved the final manuscript.
